# Hyperpolarized ^15^N-pyridine Derivatives as pH-Sensitive MRI Agents

**DOI:** 10.1038/srep09104

**Published:** 2015-03-16

**Authors:** Weina Jiang, Lloyd Lumata, Wei Chen, Shanrong Zhang, Zoltan Kovacs, A. Dean Sherry, Chalermchai Khemtong

**Affiliations:** 1 Advanced Imaging Research Center, University of Texas Southwestern Medical Center, 5323 Harry Hines Boulevard, Dallas, TX 75390, USA; 2 Department of Chemistry, University of Texas at Dallas, 800 West Campbell Road, Richardson, TX 75083, USA

## Abstract

Highly sensitive MR imaging agents that can accurately and rapidly monitor changes in pH would have diagnostic and prognostic value for many diseases. Here, we report an investigation of hyperpolarized ^15^N-pyridine derivatives as ultrasensitive pH-sensitive imaging probes. These molecules are easily polarized to high levels using standard dynamic nuclear polarization (DNP) techniques and their ^15^N chemical shifts were found to be highly sensitive to pH. These probes displayed sharp ^15^N resonances and large differences in chemical shifts (Δδ >90 ppm) between their free base and protonated forms. These favorable features make these agents highly suitable candidates for the detection of small changes in tissue pH near physiological values.

MR imaging agents with excellent pH-sensing capability and imaging sensitivity could become very useful tools for monitoring tissue acidosis that presents in many disease states including rapidly growing tumors^1^,^2^,^3^. Such pH-sensitive agents must have pK_a_ values near the pH of interest, ideally around ~6.7, display large pH-induced changes in chemical shift, and have a suitable MR sensitivity for imaging pH *in vivo*. Several pH-sensitive probes reported previously rely on magnetic resonance spectroscopy (MRS) for readout of pH^4^. These include ^1^H-5,^19^F-6, and ^31^P-based agents^7^,^8^. Chelated Gd(III) and Eu(III) complexes have also been developed as MRI agents for pH mapping based on pH-dependent relaxivity and chemical exchange saturation transfer (CEST) imaging^9^,^10^, respectively. Despite some success using the Gd(III)-based agents to image pH *in vivo*, these agents require a separate measure of agent concentration for pH calibration. CEST agents offer the possibility of obtaining a direct readout of pH using ratiometric methods but the image sensitivity of such agents remains problematical. Recently, it has been reported that the sensitivity of NMR insensitive nuclei can be significantly improved, >10,000-fold or more, by use of dynamic nuclear polarization (DNP) methods^11^. In DNP, the extremely high thermal polarization of free radical electrons is transferred to the nuclear spins of NMR-active nuclei, such as ^13^C and ^15^N, by microwave irradiation of a frozen sample. The transfer generates a non-equilibrium “hyperpolarized” (HP) spin state with dramatically improved NMR sensitivity that returns to thermal equilibrium as a function of spin-lattice relaxation time, *T_1_*. DNP has played a key role in following real-time metabolism of HP ^13^C-labeled substrates such as [1-^13^C]pyruvate in tumors, heart, and liver^12^,^13^,^14^,^15^,^16^. Hyperpolarized chemical probes that can in real-time monitor the pH, H_2_O_2_ and redox state have also been investigated^17^,^18^,^19^. For example, Gallagher *et al* recently demonstrated that HP ^13^C-enriched bicarbonate (HP H^13^CO_3_^−^) can be used to image pH of tumors in mice^15^,^20^. The same principle was later applied to measure intracellular pH (pHi) in isolated perfused hearts, based on intracellular generation of HP H^13^CO_3_^−^/^13^CO_2_ by oxidation of HP [1-^13^C]pyruvate^21^. It is important to point out that the pH estimated using HP bicarbonate likely reflects a combination of intracellular (pHi) and extracellular pH (pHe) because CO_2_ can freely diffuse through cell membranes^20^. The short *T_1_* of H^13^CO_3_^−^ is also a potential drawback for clinical translation of exogenous HP H^13^CO_3_^−^. In another example, Jindal *et al* reported HP ^89^Y-complexes as potential agents for chemical shift imaging (CSI) of pH by MRI^22^. Potential drawbacks for these complexes include the low γ of ^89^Y (low imaging sensitivity) and the relatively small pH-dependent chemical shifts (Δ*δ*~10 ppm from pH 5 to 9) observed for such systems.

Herein, we demonstrate the potential utility of using HP ^15^N-pyridine derivatives as ultrasensitive pH imaging agents (Fig. 1). We first investigated the ^15^N NMR signal enhancement by DNP of the sp^2^-hybridized nitrogens in pyridine derivatives (i.e. pyridine, 2,6-lutidine, 2-picoline, nicotinamide, and 2,4,6-collidine) and the ^15^N chemical shift pH dependence. Previously, it has been shown that the ^15^N chemical shifts of certain nitrogen functional groups were highly sensitive to protonation and a sharp chemical shift change near the pKa was observed^23^. Combining the strong pH-dependent chemical shift with the potentially substantial signal enhancement by DNP, HP ^15^N-enriched agents could be ideal candidates for chemical shift imaging of tissue pH. So far, hyperpolarization of ^15^N-labeled compounds by DNP has been demonstrated with significant ^15^N NMR signal enhancement^11^,^24^. It has also been shown that some ^15^N-labeled functional groups such as ^15^N-enriched choline (~4 min)^24^, and ^15^N-enriched nitro compounds (~100 s)^25^ have much longer *T_1_* than carbonyl ^13^C centers.

## Results and Discussion

### ^15^N-NMR signal enhancement and spin-lattice relaxation time of pH probes

**Figure 2:**
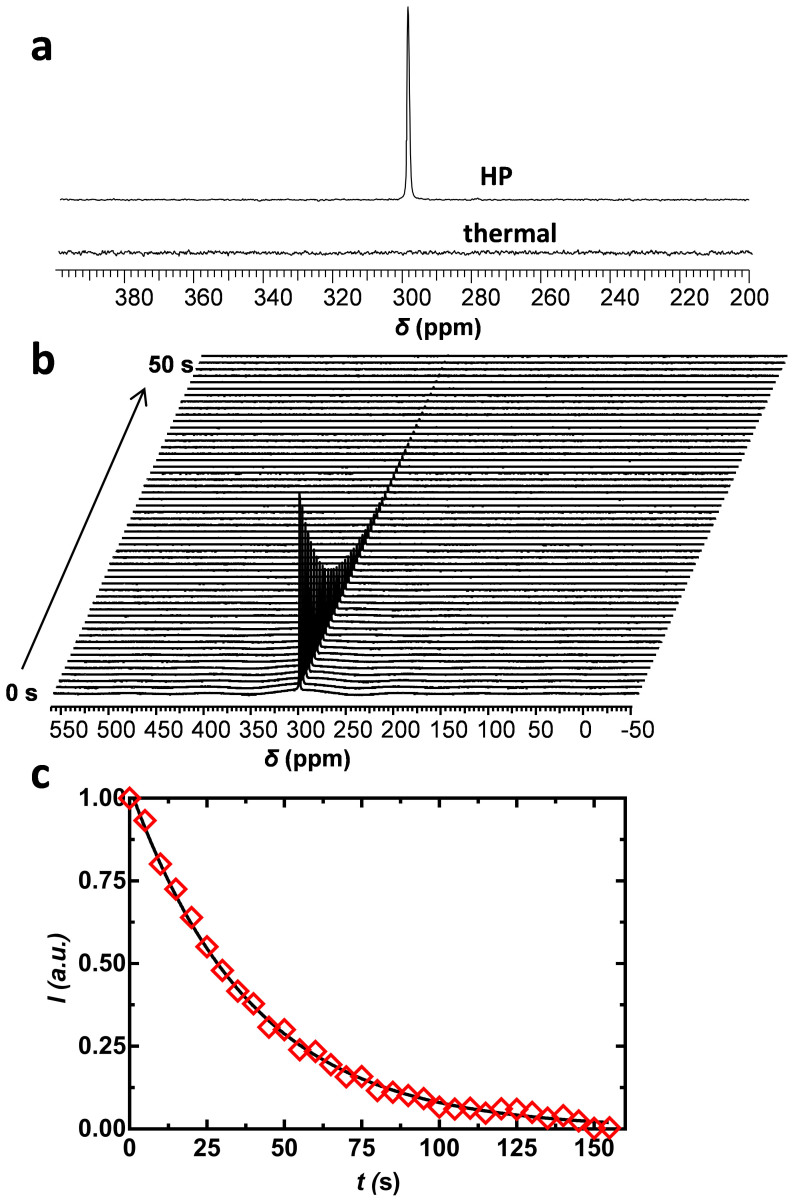
(a) ^15^N NMR spectra of a HP (*top*) and thermal (*bottom*) sample of ^15^N-pyridine. (b) array of ^15^N NMR of HP ^15^N-pyridine after dissolution in water; (c) Representative *T_1_* decay of HP ^15^N-pyridine in water.

In this study, we aim to develop HP spectroscopic imaging agents that are highly sensitive to pH changes. Pyridine derivatives were chosen for evaluation because it is known that their ^15^N resonances have relatively long *T_1_*'s and are highly pH-sensitive^23^. The pKa values of pyridine derivatives are optimal for tissue pH measurements and can also be altered considerably by adding substituents. Therefore, it is highly possible that pyridine derivatives having suitable pKa values for any specific pH measurement applications can be designed and synthesized. Despite the excellent sensing capability, the role of ^15^N NMR in molecular imaging is very limited mainly due to its poor sensitivity. To overcome such shortcoming, we investigated DNP hyperpolarization as a means to improve the ^15^N NMR sensitivity of pH-sensitive pyridine derivatives. Our results showed that these pyridine derivatives can be well polarized by DNP with high ^15^N signal enhancements. After dissolution into water, the ^15^N signal enhancements of 5437 ± 200, 8702 ± 700, 7877 ± 400, 10065 ± 600, and 3598 ± 700 were observed for ^15^N-pyridine, ^15^N-2,6-lutidine, ^15^N-2-picoline, ^15^N-2,4,6-collidine and ^15^N-nicotinamide, respectively. This level of enhancement is comparable to previously reported values for aromatic ^15^N compounds^25^. The observed higher polarization of methyl-substituted pyridine compounds was likely due to their greater tendency to form frozen glasses when mixed with the DMSO/Sulfolane glassing agent^25^. ^15^N NMR spectra of hyperpolarized and thermal samples of ^15^N-pyridine are compared in Fig. 2a. Here, the ^15^N NMR spectra of the HP sample was collected from a single 10-degree pulse while the thermally polarized spectra represent a total signal from 256 scans using 90-degree pulses. It is important to note that both the HP and thermally polarized spectra were acquired from the same sample. The big difference in signal intensity demonstrates the tremendous signal enhancement achieved by DNP hyperpolarization.

**Figure 3:**
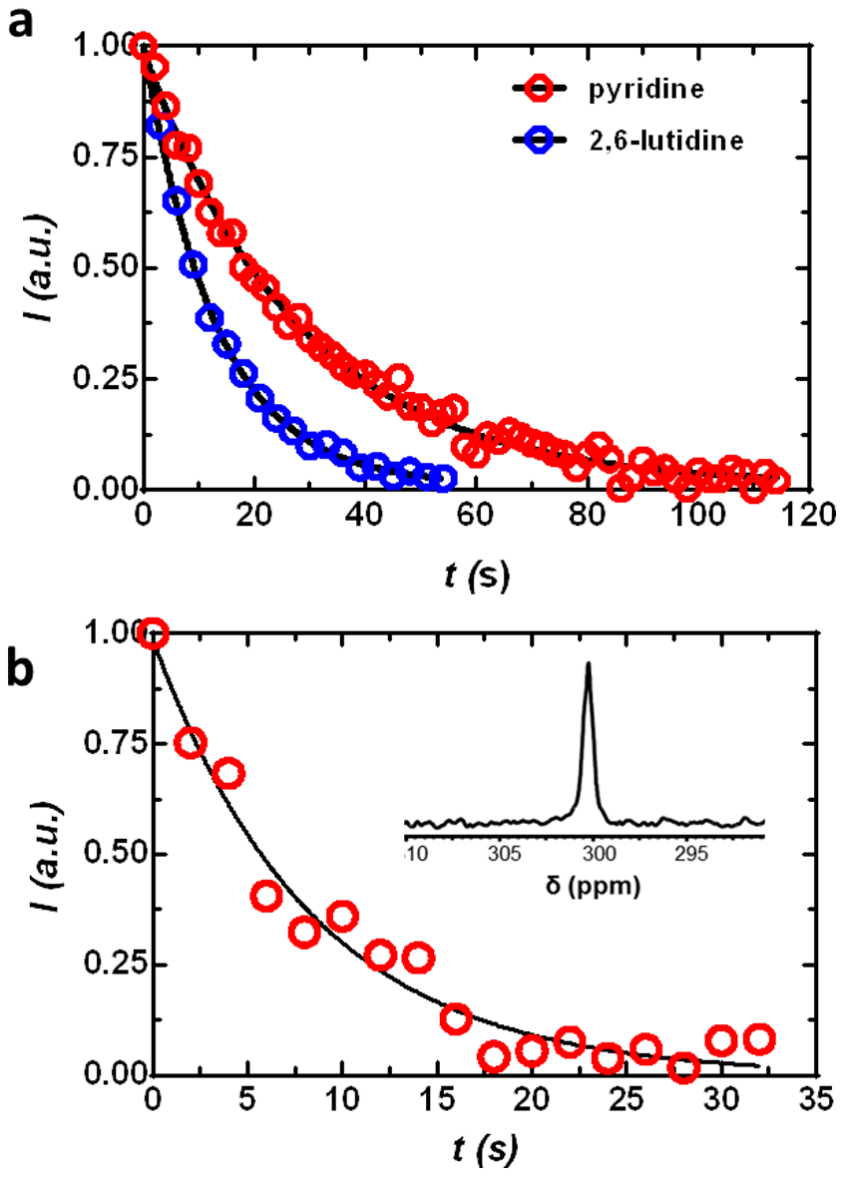
(a) Representative *T_1_* decays of HP ^15^N-pyridine and ^15^N-2,6-lutidine at physiological pH; (b) and HP ^15^N-pyridine in rat plasma. The inset shows ^15^N NMR spectrum of HP ^15^N-pyridine in plasma obtained by summing the ^15^N signal over 10 scans.

Fig. 2b shows an array of representative ^15^N NMR spectra of HP ^15^N-pyridine collected every 5 s using a 5-degree pulse after dissolution with water. By fitting such time-dependent ^15^N signal intensity decay curves (Fig. 2c), the *T_1_* of HP ^15^N-pyridine (pH 8.4), ^15^N-2,6-lutidine (pH 9.5), ^15^N-2-picoline (pH 8.5), and ^15^N-nicotinamide (pH 5.9) were found to be 41 ± 3 s, 31 ± 2 s, 38 ± 2 s and 22 ± 0.3 s, respectively (Supplementary Fig. S1 online). Due to poor water solubility, dissolution of 2,4,6-collidine required the use of methanol. Here, the measured *T_1_* was 36 ± 4 s. The presence of methyl substituents on the pyridine ring results in a larger number of protons in close proximity to the nitrogen which results in more efficient dipole-dipole relaxation of the pyridyl ^15^N. Nevertheless, these *T_1_* values are comparable to many ^13^C carboxyl groups at this same field currently used for metabolic imaging. The hyperpolarized ^15^N signal of pyridinyl derivatives decayed more rapidly near physiological pH (Fig. 3a), likely reflecting an added relaxation pathway by the exchanging protons as these molecules become partially protonated^26^. As expected, the *T_1_* of ^15^N-pyridine measured in plasma at 37°C was shorter (~11 s, Fig. 3b). While undesirable, this should not prevent the *in vivo* application of ^15^N-labeled pyridine derivatives as pH probes because protonation equilibria are established nearly instantaneously. In comparison, the ^13^C bicarbonate/carbon dioxide equilibrium system has been used successfully for imaging tissue pH *in vivo* even though the *T_1_* of the ^13^C atoms in these molecules were only ~10 s^20^.

### pH sensitivity of HP ^15^N-pyridine derivatives

**Figure 4:**
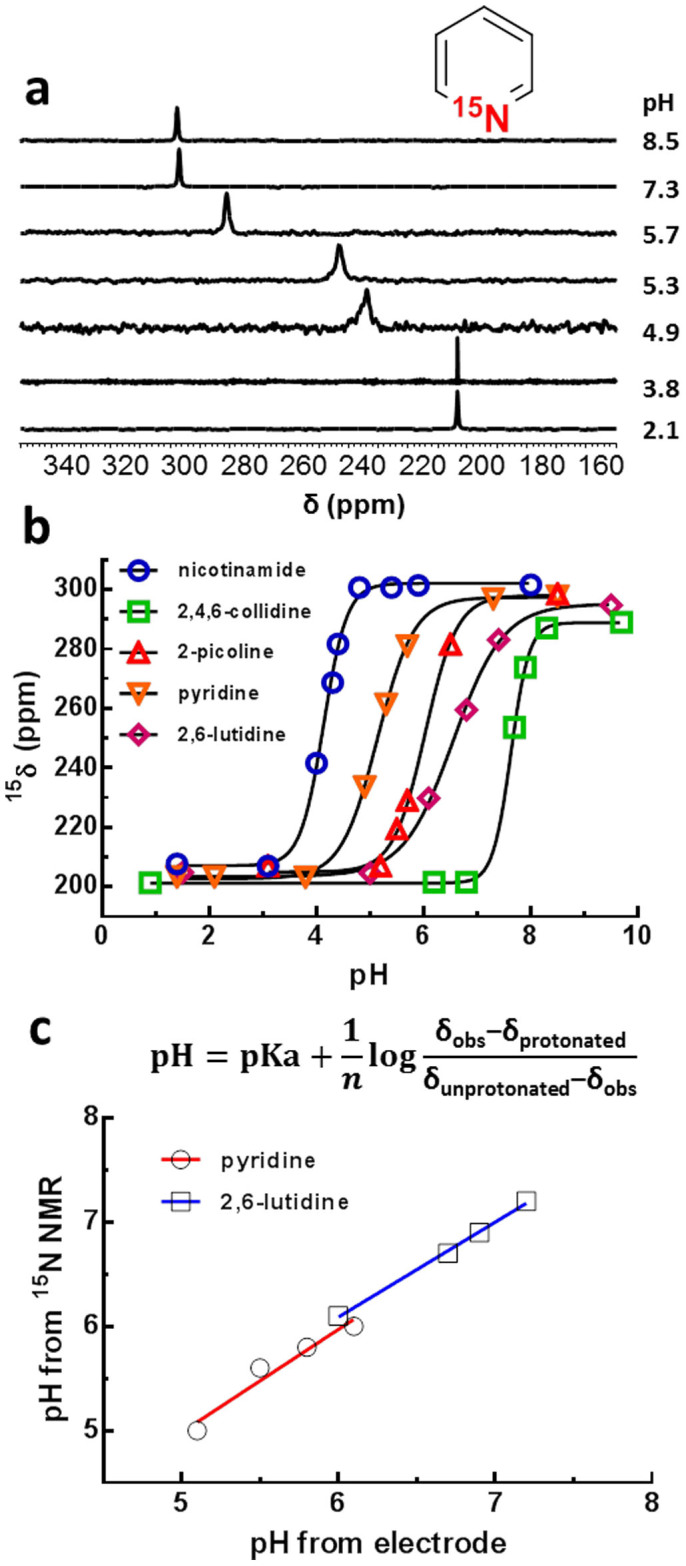
^15^N chemical shift versus pH for HP ^15^N-agents; (a) ^15^N-NMR spectra of hyperpolarized ^15^N-agents versus pH; (b) ^15^N-NMR titration curves of hyperpolarized ^15^N-agents; (c) correlation of pH calculated from the Hendersen-Hasselbalch equation (displayed above) and pH electrode. δ_obs_ is the ^15^N chemical shift observed from NMR spectra, δ_protonated_ is the ^15^N chemical shift of fully protonated pyridine, δ_unprotonated_ is the ^15^N chemical shift of basic pyridine, n is the Hill coefficient.

^15^N NMR has long been known to have a great sensing capability for different chemical environments such as acidity^23^ and metal ion concentrations^27^. Protonation or metal chelation of some nitrogen-containing compounds can lead to a significant change in ^15^N chemical shift, making them suitable MR sensors for pH or metal ions. For pyridine derivatives, the electronic properties of the *sp*^*2*^-hybridized, aromatic nitrogen center are greatly altered upon protonation leading to a significant change in ^15^N chemical shift. ^15^N NMR spectra of HP ^15^N-pyridine samples adjusted to different pH values are shown in Fig. 4a. All five ^15^N-pyridine derivatives shift upfield upon protonation (Fig. 4b). The ^15^N NMR linewidth of each probe was narrow in both the fully protonated or fully deprotonated forms but broaden somewhat at pH values near their respective pK_a_'s. This broadening reflects intermediate rates of exchange of protons between the ^15^N atom and water^26^. The pK_a_ values estimated by fitting the pH titration curves were 5.17 ± 0.07 for pyridine, 6.60 ± 0.02 for 2,6-lutidine, 6.02 ± 0.05 for 2-picoline, 7.65 ± 0.05 for 2,4,6-collidine, and 4.14 ± 0.02 for nicotinamide. These pKa values agreed well with values previously reported^28^. The ^15^N chemical shift differences between the free base and fully protonated forms were all in the range, 88–94 ppm (Table 1). The chemical shifts of all five ^15^N enriched compounds changed linearly with pH at least over ±1 pH units of each ligand pK_a_. The data demonstrate that these ^15^N probes are quite sensitive to changes in pH (~60 ppm/pH unit) and that structures can be modified to fine-tune the probe to readout any desired pH value from 5 to 8.5.

To test the accuracy of these pH probes, a series of samples containing HP ^15^N-pyridine or ^15^N-2,6-lutidine with variable amounts of HCl added to the dissolution solution were examined by ^15^N-NMR (Supplementary Fig. S2 online). The two agents were chosen because of their long *T_1_* values. For each pH probe, the test was carried out over a pH range near the respective pKa values where the ^15^N chemical shifts are extremely sensitive to pH. The pH of each sample was estimated from the observed ^15^N chemical shifts (Fig. 4b) and also measured using a pH electrode^29^. Fig. 4c shows plots of pH as measured by ^15^N-NMR versus a pH electrode. The results show an excellent correlation between the two measurements for both probes (R^2^ > 0.99 for 2,6-lutidine and R^2^ > 0.95 for pyridine), demonstrating the high accuracy of pH measurements by ^15^N NMR of HP-pyridine derivatives.

### Magnetic resonance spectroscopic imaging of HP ^15^N-Pyridine

**Figure 5:**
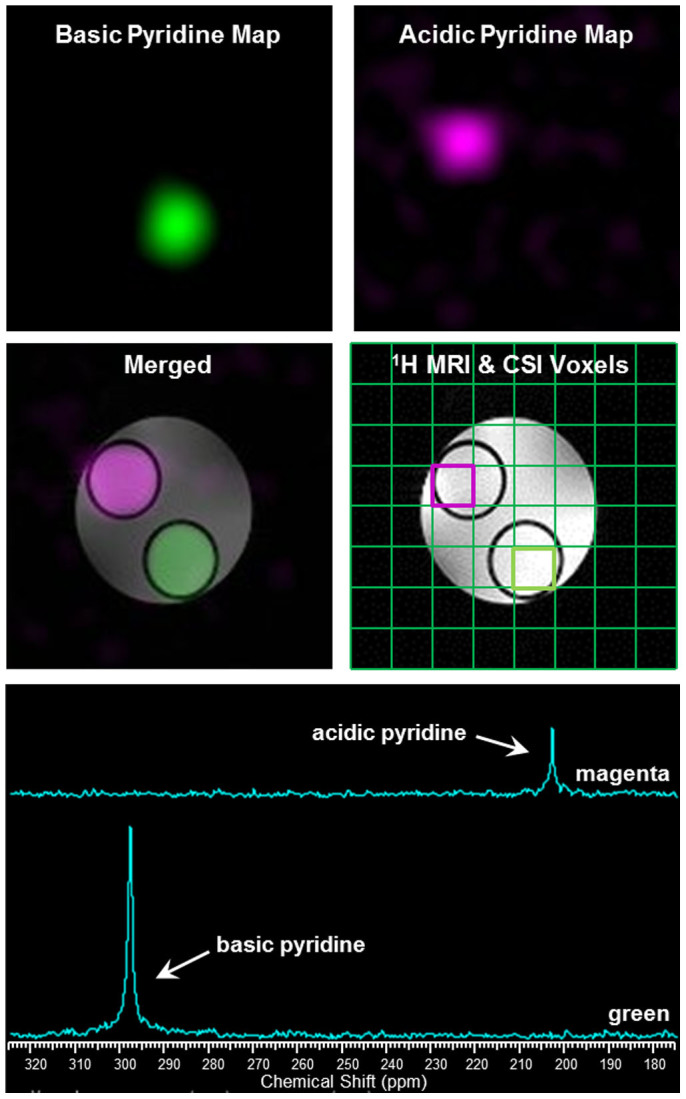
^15^N MR images of HP-pyridine in basic (*top left*) and protonated (*top right*) forms. Localization of the ^15^N images to the^1^H reference image is shown in the merged image.^1^H MRI of a phantom showing CSI grids is shown in the middle right panel and the ^15^N NMR spectra of highlighted voxels are shown in the bottom panel.

Chemical shift images of phantoms containing HP ^15^N-enriched pyridine were collected to test the applicability of applying HP ^15^N-pyridine derivatives to distinguish pH differences. In this experiment, a solution of HP ^15^N-pyridine was simultaneously injected into two NMR tubes inserted within a large NMR tube (see Supplementary Fig. S3 online for the phantom setup). One of the small tubes contained a predetermined amount of sulfuric acid while the other contained sodium hydroxide. The imaging plane was positioned axially covering both tubes of HP-pyridine solutions (see^1^H image in Fig. 5). ^15^N CSI data demonstrate that the ^15^N signals of both basic and acidic HP-pyridine compartments were detectable by MRI. ^15^N NMR spectra of selected voxels show ^15^N resonances at 297.4 and 207.5 ppm reflecting the free base and protonated pyridine samples, respectively. CSI images of both basic and acidic pyridine displayed good localization of these two signals within the tubes (see CSI and merged image). The results show that magnetic resonance spectroscopic imaging (MRSI) of HP ^15^N-enriched pyridine was able to distinguish different pH environments in adjacent spatial locations in a phantom. It is worth noting that the imaging of HP ^15^N-pyridine was very rapid and a similar image of thermally polarized ^15^N-enriched pyridine would have required a much higher ^15^N spin concentration and a longer imaging time. These results strongly emphasize the sensitivity advantage of HP ^15^N for molecular imaging applications over thermally polarized imaging practices. To the best of our knowledge, these results demonstrate for the first time the feasibility of ^15^N spectroscopic imaging of pH-sensitive agents, strongly emphasizing the sensitivity advantage of HP ^15^N for molecular imaging applications.

Potential *in vivo* imaging applications of these agents include, but not limited to, pH imaging or MR spectroscopic assessment of tissue acidosis that is present in many diseases. However, many considerations must be taken into account before HP pyridine derivatives can be translated for *in vivo* evaluations. First, the HP pyridine agents should preferably be present as a free base or be partially protonated at the physiological pH. The long *T_1_* of the free base will allow for the HP signal to be retained much longer during the pre-injection quality controls of the agents. Additionally, the pH regions of the targeted tissues should not be very close to the pKa of the agents in order to avoid the rapid signal loss from fast water proton exchanges. Therefore, the ideal HP pyridine derivatives should have a pKa that is slightly below the pH of acidic tissues to be assessed in order to provide a relatively large chemical shift window and relatively long HP signal lifetime. The toxicity of these agents is also an important aspect that needs to be considered. Although the toxicity of pyridine is a concern, there are several naturally occurring pyridine derivatives that can be considered. Some of these compounds are present in the biological systems and play key roles in human physiology. For example, nicotinamide is a building block for nicotinamide adenine dinucleotide (NAD) and nicotinamide adenine dinucleotide phosphate (NADP) while nicotinic acid (vitamin B_3_) and pyridoxyl derivatives (vitamin B_6_) are essential human nutrients. Another derivative, picolinic acid, is a product of the kynurenine pathway and is a neuroprotective compound^30^. Chemical modifications of these molecules will be required for potential *in vivo* pH imaging applications to alter their pKa values toward the desirable range while preserving their biocompatibility. Future work will be focused on biocompatible pyridinyl compounds with favorable pK_a_ values for pH imaging of tissue acidosis.

## Conclusion

We have demonstrated the potential of using HP ^15^N-pyridine derivatives as pH-sensitive probes for MRI. These molecules display large changes in ^15^N chemical shift with pH and have very sharp chemical shift versus pH titration curves. The combination of hyperpolarization and pH-sensitive ^15^N agents offers new opportunities to develop highly sensitive imaging agents with great sensing capability for the characterization of important biomarkers such as tissue acidity. Future work will be focused on *in vivo* pH imaging of acidic tumors.

## Methods

### Acquisition of ^15^N-NMR spectra of hyperpolarized ^15^N-agents

Unless otherwise noted, all chemicals and solvents were purchased from Sigma-Aldrich (St. Louis, MO, USA) and used as received. A mixture of pyridine (6.2 M), 2,6-lutidine (4.3 M), 2-picoline (5.0 M), nicotinamide (2.7 M) or 2,4,6-collidine (3.78 M) and BDPA radical (40 mM) dissolved in 50 μL of DMSO-sulfolane (1:1 v/v) was polarized in a HyperSense polarizer (3.35 T, Oxford Instruments Molecular Biotools, UK) according to the manufacturer's procedures. The polarization was carried out at ~1.05 K with 94.055 GHz microwave irradiation for 2 h. The dissolution liquid of HP agents in distilled water (4 mL) from the polarizer was rapidly mixed with a pre-determined volume of hydrochloric acid or sodium hydroxide in a 10-mm NMR tube to achieve the desired pH. ^15^N-NMR spectra were acquired at room temperature (~23 °C) on a 400-MHz spectrometer using a 5 degree flip angle with a repetition time (TR) of 5 s. All ^15^N peaks were externally referenced with ^15^N-nitrobenzene (372 ppm). ^15^N MRI were acquired on a 9.4 T Agilent vertical bore microimager (Agilent, USA). MR images and spectra were processed using ImageJ (NIH, USA) and ACD/SpecManager (ACD Labs, Canada). The *T_1_,* signal enhancement and pH titration experiments were investigated with the natural abundant ^15^N compounds. ^15^N-labeled pyridine was used in *T_1_* measurement in plasma and CSI experiments.

### *T_1_* relaxation times and signal enhancements of HP pyridine derivatives

In a typical protocol, a dissolution liquid (4 mL) of HP-pyridine derivative was rapidly transferred into a 10-mm NMR tube. ^15^N NMR acquisition was initiated once the transfer was complete. An array of ^15^N spectra with one spectra recorded every 5 s was obtained (*TR* = 5 s, flip angle = 5 degree). ^15^N signal intensity of the HP nuclei was normalized to the signal intensity of the first time point (*t* = 0 s). By fitting the NMR signal intensity as a function of time, *T_1_* values were calculated using Equation (1)^31^, where *M_0_* is the original magnetization, *TR* is the repetition time, and *θ* is the flip angle. Liquid-state ^15^N signal enhancements of HP pyridine derivatives were measured by comparing ^15^N NMR signal of HP versus thermally polarized samples. A single 5-degree pulse was acquired for the HP signal. For thermally polarized signal, a 20-mM solution of a pyridine compound was used. 256 scans of ^15^N NMR spectra were acquired using 90-degree pulses with a 500second delay (>5 times *T_1_*). Enhancement levels were calculated as ratios of the ^15^N signal from the two polarized states, taking into account for the different ^15^N concentrations and number of scans (1 *vs* 256). PBS buffer solution (pH = 7.4) was used as a dissolution solvent for *T_1_* measurements at the physiological pH. The final concentration of HP ^15^N-pyridine or 2,6-lutidine was 2 mM. The plasma used in *T_1_* of HP ^15^N-pyridine was obtained by centrifugation of whole rat blood to remove red blood cells. In this experiment, the dissolution liquid of HP ^15^N-pyridine (2 mL) was well mixed with rat plasma (2 mL) in a 10-mm NMR tube. The final concentration of HP ^15^N-pyridine was 13 mM. ^15^N-NMR spectrum was acquired at ~37°C. 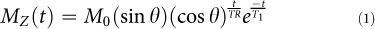


### pH titration curves

Titration curves were created by plotting ^15^N chemical shift versus pH as measured from a pH meter. For the determination of pH by HP ^15^N-NMR, HP-pyridine or HP-2,6-lutidine was mixed with an unknown amount of HCl or NaOH in an NMR tube before acquiring ^15^N-NMR spectra. pH of the solution was calculated from the apparent ^15^N chemical shift using the Henderson–Hasselbalch equation and the pKa value estimated by HP-NMR titration. The pH values obtained from HP ^15^N-NMR were plotted against the values measured by a pH electrode.

### Chemical shift imaging (CSI) of HP ^15^N-pyridine

The phantom for this experiment was a 25-mm NMR tube encasing two 10-mm NMR tubes, with one pre-added sulfuric acid (200 μL, 7 M) and another had sodium hydroxide (200 μL, 5 M). The phantom was inserted into a 25-mm NMR tube containing DI water (~10 mL). The imaging plane was positioned axially across the phantom (Supplementary Fig. S3a online). ^15^N CSI was acquired after the transfer was completed with a 10-s delay to allow for complete mixing. pH values of the HP-pyridine solutions measured by pH electrode in the small tubes were 2 and 11. ^15^N CSI parameters: CSI2d sequence (Agilent VnmrJ 4 Imaging, Agilent, USA), field of view (FOV) = 40 × 40 mm^2^; TR = 200 ms; TE = 1.30 ms; flip angle = 20°; number of average (NA) = 1. A^1^H reference image was acquired at the same slice position using a GEMS sequence.^1^H imaging parameters: FOV = 40 × 40 mm^2^; TR = 200 ms; TE = 4 ms; flip angle = 20°; NA = 1. Matrix = 8 × 8; voxel size = 5 × 5 × 15 mm^3^. The CSI data were processed to 128 × 128 matrix.

## Author Contributions

C.K. initiated and directed the project, analyzed data, and wrote the manuscript. W.J. performed the experiments, analyzed data, and wrote the manuscript. L.L., W.C., S.Z., Z.K. and A.D.S. contributed to data analyses and manuscript preparation.

## Supplementary Material

Supplementary Information Supplementary Information

## Figures and Tables

**Figure 1:**
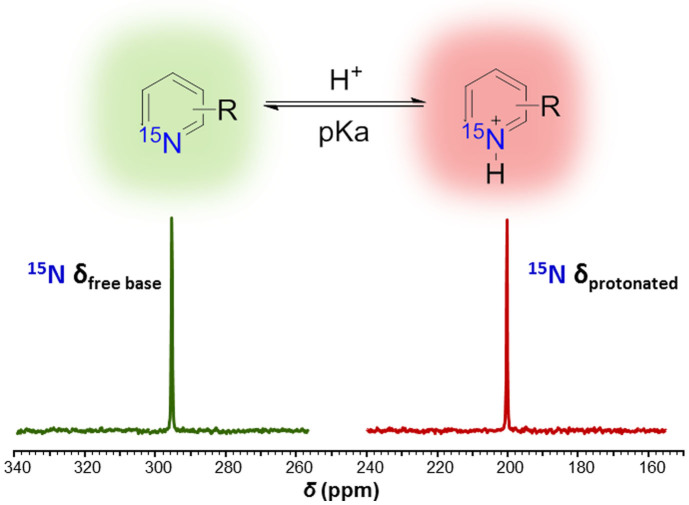
^15^N NMR spectra of HP-pyridine as the free base (*green*) and fully protonated (*red*) forms. The large chemical shift difference demonstrates that pyridine derivatives may serve as ultra-sensitive pH probes. The two spectra were acquired separately from HP-pyridine samples dissolved in either base or acid.

**Table 1:** *T_1_* relaxation time, signal enhancement, pKa, and chemical shift changes of HP ^15^N-pyridine, ^15^N-2,6-lutidine, ^15^N-2-picoline ^15^N-2,4,6-collidine, and ^15^N-nicotinamide. *T_1_*, enhancement, and pKa are reported as mean values ± SD

Compound	*T_1_*(s)[a]	Enhancement[b]	pKa[c]	^15^N Δδ(ppm)
pyridine	41 ± 3	5437 ± 200	5.17 ± 0.07	94
2,6-lutidine	31 ± 2	8702 ± 700	6.60 ± 0.02	90
2-picoline	38 ± 2[d]	7877 ± 400	6.02 ± 0.05	94
2,4,6-collidine	36 ± 2[d]	10065 ± 600	7.65 ± 0.05	88
nicotinamide	22 ± 0.3	3598 ± 700	4.14 ± 0.02	94

^[a]^*T_1_* was calculated using Equation 1;

^[b]^enhancements were measured after dissolution;

^[c]^pKa was estimated by fitting pH titration curves.

^[d]^*T_1_* of 2,4,6-collidine was estimated in methanol.
