# Supplementary material for: Hyperpolarized 15N-pyridine Derivatives as pH-Sensitive MRI Agents
Source: Sci Rep. 2015 Mar 16;5:9104. doi: 10.1038/srep09104 (PMC4360734; doi:10.1038/srep09104)
Supplement: Supplementary Information [file srep09104-s1.pdf]

Hyperpolarized ^{15}N-pyridine Derivatives as pH-Sensitive MRI Agents

*Weina Jiang, Lloyd Lumata, Wei Chen, Shanrong Zhang, Zoltan Kovacs, A. Dean Sherry & Chalermchai Khemtong**

Advanced Imaging Research Center, University of Texas Southwestern Medical Center, 5323 Harry Hines Boulevard, Dallas, TX 75390 (USA)

Address correspondence to:
Chalermchai Khemtong
Advanced Imaging Research Center
University of Texas Southwestern Medical Center
5323 Harry Hines Blvd.
Dallas, TX 75390, USA
Tel: 214-645-2772
Fax: 214-645-2744

E-mail: chalermchai.khemtong@utsouthwestern.edu

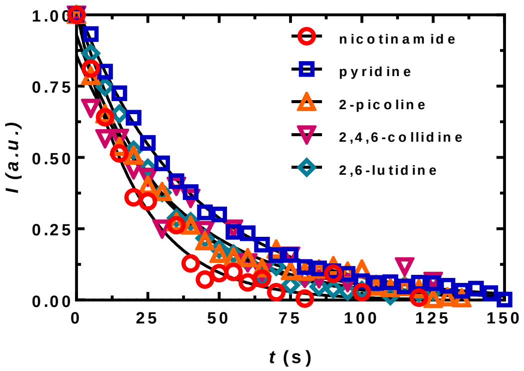

Figure S1. Decay of hyperpolarized ^{15}N-pyridine and its derivatives (TR = 5 s, flip angle = 5 degree)

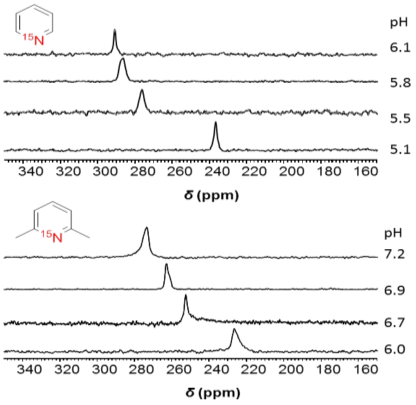

Figure S2. ^{15}N-NMR of hyperpolarized ^{15}N-pyridine and ^{15}N-2,6-lutidine with unknown amount of HCl

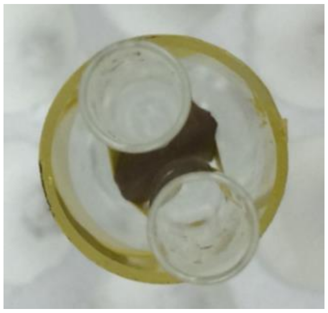

Top view

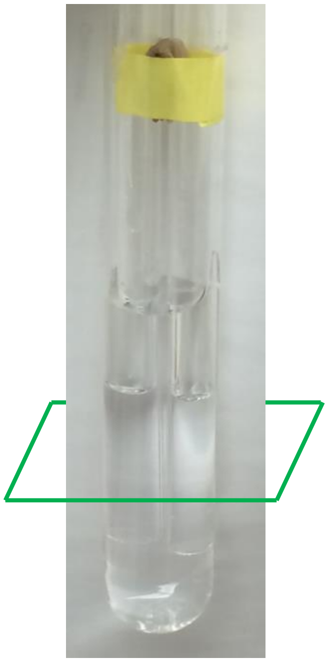

The phantom in a 25-mm NMR tube, showing the imaging slice

Figure S3. Pictures showing a phantom setup for the CSI imaging of HP ^{15}N-pyridine
